# A mechanistic spatio-temporal framework for modelling individual-to-individual transmission—With an application to the 2014-2015 West Africa Ebola outbreak

**DOI:** 10.1371/journal.pcbi.1005798

**Published:** 2017-10-30

**Authors:** Max S. Y. Lau, Gavin J. Gibson, Hola Adrakey, Amanda McClelland, Steven Riley, Jon Zelner, George Streftaris, Sebastian Funk, Jessica Metcalf, Benjamin D. Dalziel, Bryan T. Grenfell

**Affiliations:** 1 Department of Ecology and Evolutionary Biology, Princeton University, Princeton, New Jersey, United States of America; 2 Maxwell Institute for Mathematical Sciences, Department of Actuarial Mathematics and Statistics, Heriot-Watt University, Edinburgh, United Kingdom; 3 Department of Plant Sciences, University of Cambridge, Cambridge, United Kingdom; 4 International Federation of Red Cross and Red Crescent Societies, Geneva, Switzerland; 5 MRC Centre for Outbreak Analysis and Modelling, Department Infectious Disease Epidemiology, Imperial College, London, United Kingdom; 6 School of Public Health, University of Michigan, Ann Arbor, Michigan, United States of America; 7 Centre for the Mathematical Modelling of Infectious Diseases, London School of Hygiene & Tropical Medicine, London, United Kingdom; 8 Department of Integrative Biology, Oregon State University, Corvallis, Oregon, United States of America; 9 Department of Mathematics, Oregon State University, Corvallis, Oregon, United States of America; The Pennsylvania State University, UNITED STATES

## Abstract

In recent years there has been growing availability of individual-level spatio-temporal disease data, particularly due to the use of modern communicating devices with GPS tracking functionality. These detailed data have been proven useful for inferring disease transmission to a more refined level than previously. However, there remains a lack of statistically sound frameworks to model the underlying transmission dynamic in a mechanistic manner. Such a development is particularly crucial for enabling a general epidemic predictive framework at the individual level. In this paper we propose a new statistical framework for mechanistically modelling individual-to-individual disease transmission in a landscape with heterogeneous population density. Our methodology is first tested using simulated datasets, validating our inferential machinery. The methodology is subsequently applied to data that describes a regional Ebola outbreak in Western Africa (2014-2015). Our results show that the methods are able to obtain estimates of key epidemiological parameters that are broadly consistent with the literature, while revealing a significantly shorter distance of transmission. More importantly, in contrast to existing approaches, we are able to perform a more general model prediction that takes into account the susceptible population. Finally, our results show that, given reasonable scenarios, the framework can be an effective surrogate for susceptible-explicit individual models which are often computationally challenging.

## Author summary

Availability of individual-level, spatio-temporal disease data (e.g. GPS locations of infected individuals) has been increasing in recent years, primarily due to the increased use of modern communication devices such as mobile phones. Such data create invaluable opportunities for researchers to study disease transmission on a more refined individual-to-individual level, facilitating the designs of potentially more effective control measures. However, the growing availability of such precise data has not been accompanied by development of statistically sound mechanistic frameworks. Developing such frameworks is an essential step for systematically extracting maximal information from data, in particular, evaluating the efficacy of individually-targeted control strategies and enabling forward epidemic prediction at the individual level. In this paper we develop a novel statistical framework that overcomes a few key limitations of existing approaches, enabling a machinery that can be used to infer the history of partially observed outbreaks and, more importantly, to produce a more comprehensive epidemic prediction. Our framework may also be a good surrogate for more computationally challenging individual-based models.

## Introduction

**Fig 1:**
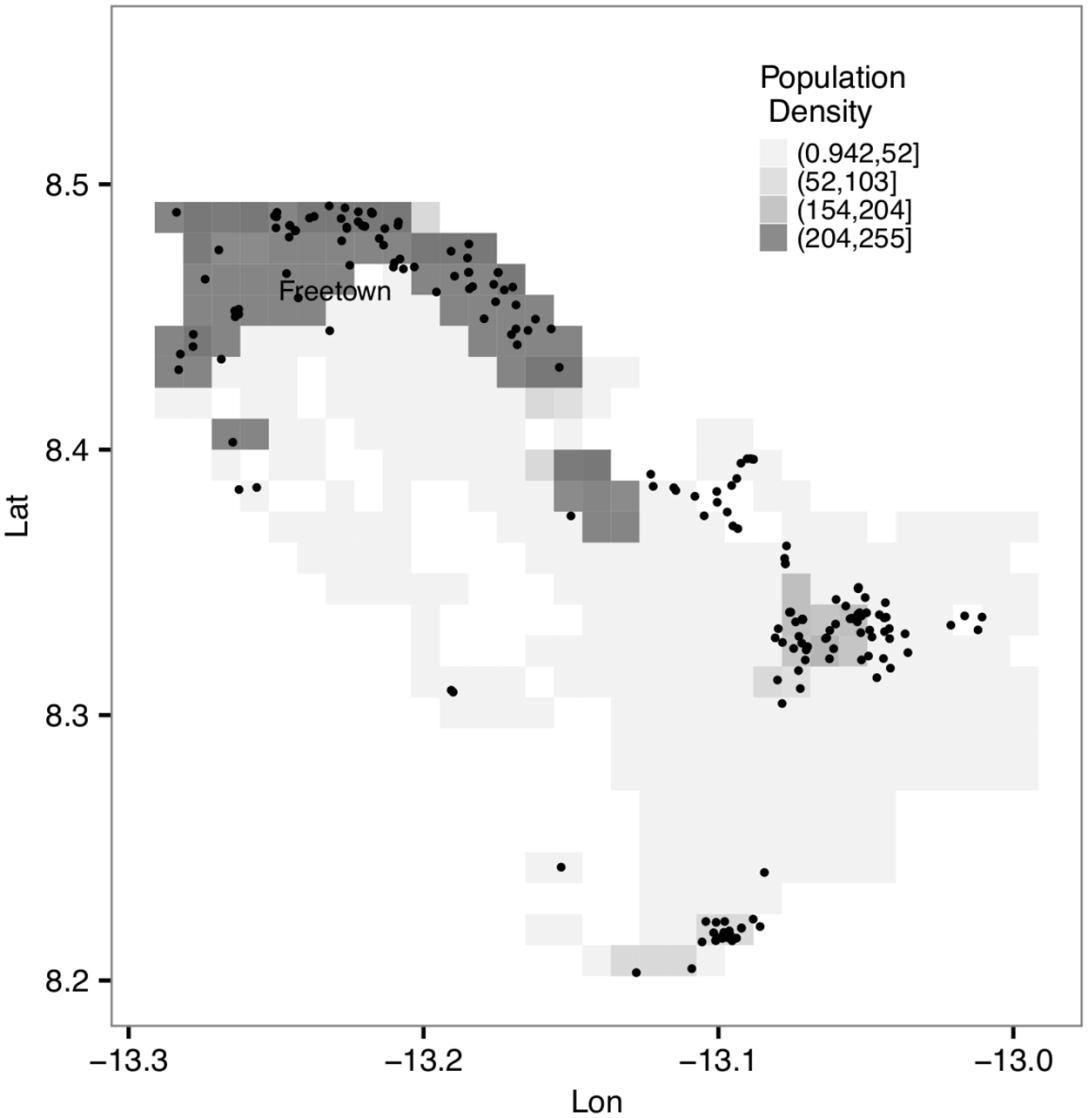
GPS locations of individual Ebola deaths (2014-2016) in the community identified through the use of mobile phones, collected in the neighbouring area of the capital (Freetown) in Sierra Leone. See the section *Ebola Outbreak Data* for more details.

Epidemiological data collected by traditional public health surveillance often contain relatively coarse spatial and temporal information on infected individuals. In recent years, the amount and resolution of the spatio-temporal data have increased vastly due to the advent of ‘digital epidemiology’ along with the increased use of modern communication devices [1], particularly through the use of mobile phones which drastically improves the tracking of human contacts [2–4]. Such data provide unprecedented opportunities for dissecting disease spread at a more localized, individual-to-individual level. The recent West Africa Ebola outbreak (Fig 1) well demonstrated the increasing availability of such data, and, in particular, the GPS location data collected during the outbreak have been shown to be useful in identifying superspreaders and quantifying the impact of superspreading during the outbreak [4].

However, the growing availability of these more precise spatio-temporal data has not been accompanied by development of statistically sound mechanistic frameworks for modelling the underlying individual-to-individual transmission process. Developing such methods is an essential step for systematically extracting maximal information from such data, in particular, evaluating the efficacy of individually-targeted control strategies and enabling forward epidemic prediction at the individual level.

**Fig 2:**
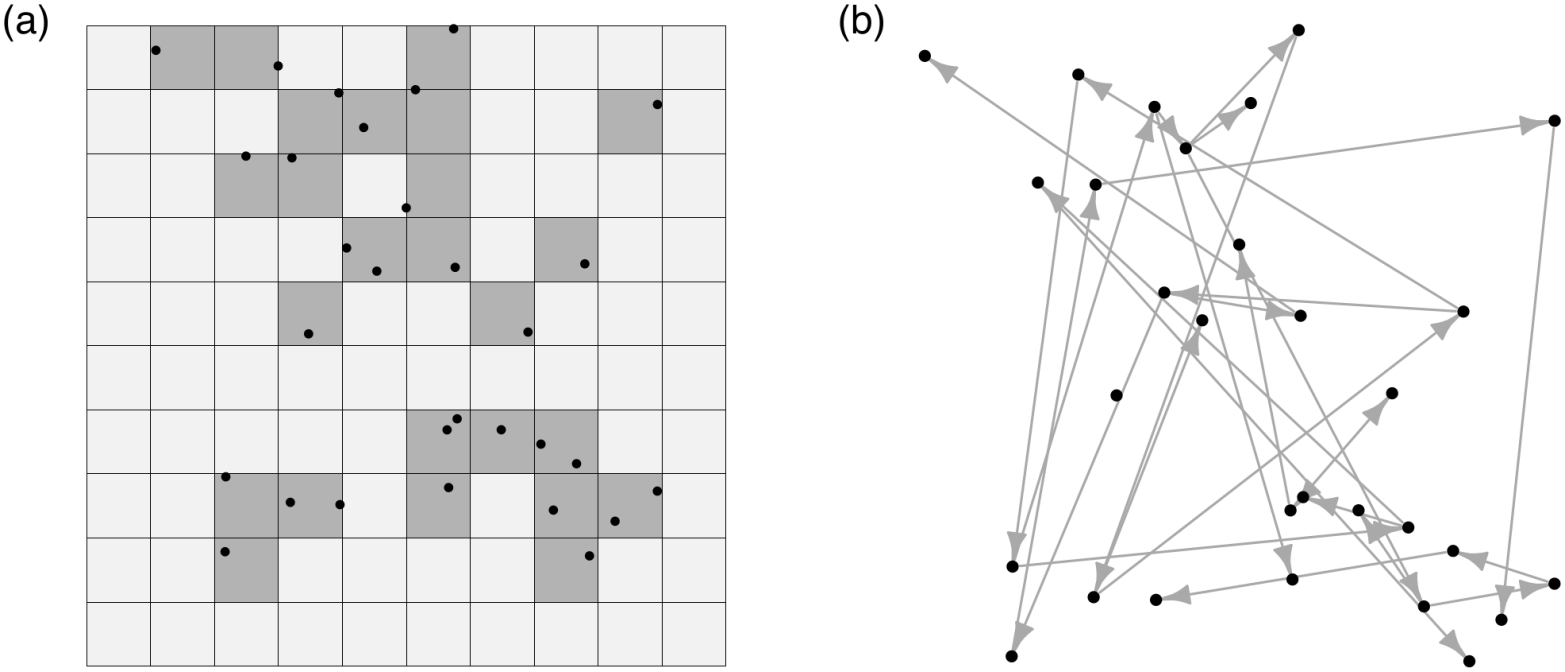
A schematic illustration of examples of existing approaches. (a) SEIR approach on patch level [7–9]. The study area is partitioned into pre-defined grids/patches, where grid-points/patches with any infected individuals (i.e. the black dots) are classified as infected (E) and grid-points without as susceptible (S). After the classification, each grid—Infected (darker gray) or Susceptible (lighter gray)—is treated as a single entity for model fitting. (b) Transmission-network-based (partial-likelihood) approach [4, 10–12]. The unknown susceptible population is completely ignored. Consider only the set of infected individuals and infer the relations, for example, the transmission path (the arrows), among them. This approach however, in contrast to the conventional SEIR model, does not delineate a mechanism of how a new infection can arise among the general (susceptible) population. Such a limitation, in particular, renders a complete forward epidemic prediction implausible.

Conventional compartmental models (e.g. SEIR) require an explicit account of the complete contact process which specifies both the successful contacts (i.e. the infected in class E), and, more challengingly [6], the unsuccessful contacts (i.e. who has remained susceptible in class S). Representing unsuccessful contacts at the individual level is computationally challenging due to the need to build an explicit contact network among essentially all individuals in the population. One may consider adapting mechanistic compartmental disease models to accommodate these data. Important examples of these approaches include: 1) a patch-level approach that aggregates data points within pre-defined grids/patches [7–9], and 2) a transmission-network-based approach which is essentially a partial-likelihood approach that considers only the infected individuals and ignores the unsuccessful contacts [4, 10–12]. Fig 2 presents a schematic illustration of these two approaches. Although the patch-level approach conforms to the desirable SEIR-type mechanistic framework, in which both the successful infectious contacts (E) and unsuccessful contacts (S) are represented, at least on the patch level, the aggregation of data points can be arbitrary and it inevitably degrades the data resolution necessary for inferring, for example, the individual-to-individual transmission. The transmission-network-based (partial-likelihood) approach, on the other hand, preserves the ‘point nature’ of the data but fails to conform to the mechanistic framework by completely ignoring the general (susceptible) population and its relation to the infected class. Although the latter has been shown to be useful for sampling the relations among infections (e.g. the transmission tree), it is inadequate for the purposes of complete forward epidemic prediction which needs to take into account the general (susceptible) population [4].

Spatio-temporal point processes (see an introduction in [5]) may also appear to be natural candidates for individual spatial data. However, it is not straightforward to integrate them with a mechanistic compartmental disease model such as the SEIR (Susceptible-Exposed-Infectious-Recovered) model. In particular, it is difficult to formulate conditional intensities for a spatio-temporal point process directly for the observations that respects the mechanistic modelling assumptions. If one observes the transitions made by individuals from the E to I classes and from the I to R classes then it may be natural to consider a *marked* spatio-temporal point process where points represent the transitions from E to I and marks quantify the subsequent sojourn time in the I class. Calculation of intensities conditional on the observation history, necessary for the construction of the likelihood, is difficult due to the transitions from E to I being unobserved. Other approaches which do not utilize the full likelihood (e.g., contact-type partial-likelihood approach [13, 14] and likelihood-free ABC approach [15]) may also be pursued. There also have been advances for more efficient parameters inference of certain classes of spatial models—for example, [16] proposes a double Metropolis-Hastings sampler for certain spatial models with intractable normalizing constants. Nevertheless, there is still a need of developing new statistical frameworks which allow for both full-likelihood-based model inference and, importantly, a statistically and biologically interpretable forward-prediction machinery that naturally integrates with mechanistic disease models and the general susceptible population.

In this paper, we develop a framework that aims to accommodate individual-level spatio-temporal data, both in a mechanistic manner and accounting for the general (susceptible) population. The approach taken can be viewed as being rooted in spatio-temporal point processes. In essence, we view the process of transmission (transitions from S to E) as a marked spatio-temporal point processes where the marks are *bivariate* and specify the subsequent sojourn times in the E and I classes for the respective exposed individual. For this formulation the conditional intensity becomes tractable as described in *Model and Methods*. We then exploit ideas that are standard in Bayesian computation—in particular data augmentation—to accommodate the lack of observation of transmission events.

We focus on epidemic outbreaks that are mainly attenuated by a time-varying transmissibility e.g. due to controls or seasonal changes of transmissibility, which is also the case for the recent West Africa Ebola outbreak [17, 18]. We also allow the occurrence of infections to be moderated both by the distance dependency of spatial infectivity and the effect of spatially heterogeneous (susceptible) population density. Such a framework enables a machinery that can be used to infer system parameters from the history of outbreaks and, more importantly, to predict the future dynamics of an epidemic. Our work represents a key generalization and extension of the work in [4, 19], notably by accounting for the effect of heterogeneous population density and considering a broader class of disease models.

Our methodology is first tested using simulated examples. We also compare our framework with the conventional, and often computationally challenging, individual-based SEIR model (which takes into account each individual in the population explicitly). Finally, it is applied to the Ebola outbreak data (Fig 1 and *Ebola Outbreak Data*), demonstrating its relevance to realistic epidemics of major current importance.

## Models and methods

### The mechanistic transmission model

We model spatio-temporal transmission, in continuous time and space and over a heterogeneous landscape with varying population density. The framework we apply to model transmission is closely related to the contact distribution model [20]. Consider the situation where there are *n*(*t*′) infectious individuals at time *t*′ among an entirely susceptible population. A new infection occurs as the first event in a non-homogeneous Poisson process with a time-varying rate *n*(*t*′) × *β*(*t*) with

$$\begin{array}{c} \beta (t)=\beta \times \exp(-\omega t), \end{array}$$ (1)

for *t* ≥ *t*′, where *β* represents the baseline transmissibility (i.e. the baseline intensity) of an infectious individual in the absence of control measures. Multiple-level baseline transmissibility *β*_*i*_, *i* = 1, 2, … may also be considered, for example, to represent heteregeneous transmissibility among different age groups (see later *Example: Application to the Ebola Outbreak Data*). The parameter *ω* quantifies the efficacy of controls that serves to reduce disease transmissibilty [21, 22]. Note that primary/background infection can be accommodated by adding a permanent infectious source presenting an additional rate *α* (i.e. the total Poisson rate becomes *α* + *β* × exp(−*ωt*)).

The source of infection of the newly infected/exposed individual is randomly chosen from the *n*(*t*′) infectious individuals. It is assumed that the probability of the new infection being at a certain distance *r* and direction *θ* away from the source of infection, is determined by the movement patterns of infectious individuals and the density of the susceptible population. Specifically, *G* = (*r*, *θ*) is drawn from a density,

$$\begin{array}{c} g\left(G;\eta ,\hat{s}\right)=f\left(r;\eta \right)\times h\left(\theta |r,\hat{s}\right), \end{array}$$ (2)

where $\hat{s}$ is the population density across the study area. Following Eq 2, the distance *r* is first drawn from *f*(*r*; *η*), a monotonically decreasing density function that specifies the likelihood of spatial movement over distance [23–25]. Specifically, we assume *r* follows an Exponential(*η*) distribution, i.e.,

$$\begin{array}{c} f(r;\eta )=\eta \times \exp(-\eta r). \end{array}$$ (3)

Given *r*, the position of the new infection is determined by a subsequent random draw *θ* from $h(\theta |r,\hat{s})$, the density of *θ* corresponding to the circle with radius *r* centered at the source of infection. When population density is homogeneous, *θ* may be drawn uniformly from 0 to 2*π*—i.e., given the homogeneous population density, there is no *a* *priori* belief that one part of the circle (i.e. the arc) is more susceptible to the occurrence of new infection than another. We consider a more general scenario with varying population density $\hat{s}$. A natural approach in specifying $h(\theta |r,\hat{s})$ is to use the population density along the circumference of the circle, denoted by $\sigma (l|r,\hat{s})$, to account for the effect of heterogeneous landscape, so that

$$\begin{array}{c} \int_{0}^{\theta^{\prime }}h\left(\theta |r,\hat{s}\right)d\theta =\int_{0}^{l^{\prime }}\sigma \left(l|r,\hat{s}\right)dl, \end{array}$$ (4)

where *l*′ is the arc length corresponding to an arbitrary angle *θ*′. It is noted that, when the source of infection is the primary/background, *r* and *θ* become irrelevant, and $g(G;\eta ,\hat{s})$ reduces to the (normalized) population density so that the probability of the new infection occurring in a neighbourhood of a particular point is proportional to the population density at that position.

Subsequently, the new infected individual is assumed to spend random times in classes *E* and *I* which are modelled using an appropriate distribution such as a Gamma or a Weibull distribution. Specifically, following [4], we use a *Gamma*(*γ*, λ) with mean *γ* and s.d. λ for the random time *x* spent in class *E*, and for the random time *x* spent in class *I* we use an $Exponential(\frac{1}{\varphi })$ with mean *φ* [4]. All sojourn times are assumed independent of each other given the model parameters.

In S1 Text, we also provide a concise description of the algorithm for simulating from the described model.

### Complete-data likelihood

Let *T* be the duration of the observation period, and let *χ*_*E*_ ⊆ *χ*_*I*_ ⊆ *χ*_*R*_ denote the sets of individuals who have entered class *E*, class *I* and class *R* by *T* respectively. Also, let **E** = (…, *E*_*j*_, …) denote the exposure times for *j* ∈ *χ*_*E*_, **I** = (…, *I*_*j*_, …) denote the times of becoming infectious for *j* ∈ *χ*_*I*_ and **R** = (…, *R*_*j*_, …) denote the times of recovery or removal for *j* ∈ *χ*_*R*_. The densities of the sojourn times in class *E* and class *I* are denoted by *f*_*E*_ and *f*_*I*_ respectively, with their corresponding cumulative distribution functions denoted by *F*_*E*_ and *F*_*I*_. Also, as previously defined, *n*(*t*) is the total number of infectious individuals at time *t*. Finally, for *j* ∈ *χ*_*E*_, let ***ψ*** = (…, *ψ*_*j*_, …) denote the collection of sources of infection for infected individuals, and **G** = (…, *G*_*j*_, …) denote their positions relative to the sources of infections where *G*_*j*_ = (*r*_*j*_, *θ*_*j*_).

Assuming complete data ***z*** = (**E**, **I**, **R**, **G**, ***ψ***) and model parameters **Θ** = (*α*, *β*, *γ*, λ, *φ*, *η*, *ω*), we can express the likelihood as

$$\begin{array}{c} \begin{array}{cc} L(\Theta ;z)= & \exp\{-\int_{0}^{T}\left(\alpha +n\left(t\right)\beta \left(t\right)\right)dt\}\\ & \times \prod_{j\in \chi_{E}^{\left(-1\right)}}P\left(j,\psi_{j}\right)\times g\left(G_{j};\eta ,\hat{s}\right)\times \left(1/r_{j}\right)\\ & \times \prod_{j\in \chi_{I}}f_{E}\left(I_{j}-E_{j};\gamma ,\lambda \right)\times \prod_{j\in \chi_{R}}f_{I}\left(R_{j}-I_{j};\varphi \right)\\ & \times \prod_{j\in \chi_{E\backslash I}}\left\{1-F_{E}\left(T-E_{j};\gamma ,\lambda \right)\right\}\times \prod_{j\in \chi_{I\backslash R}}\left\{1-F_{I}\left(T-I_{j};\varphi \right)\right\} \end{array} \end{array}$$ (5)

Here $\chi_{E}^{\left(-1\right)}$ denotes *χ*_*E*_ with the earliest exposure excluded. The contribution to the likelihood arising from the infection of *j* by the particular source *ψ*_*j*_ is given by

$$\begin{array}{c} \begin{array}{c} P\left(j,\psi_{j}\right)=\{\begin{array}{cc} \alpha , & \text{if}\;\text{individual}\;j\;\text{is}\;\text{a}\;\text{primary/background}\;\text{case,}\\ \beta (E_{j}), & \text{if}\;\psi_{j}\;\in \chi_{I}\;\text{at}\;\text{time}\;E_{j}\text{.} \end{array} \end{array} \end{array}$$ (6)

The first two lines in Eq 5 together represent the contribution to the likelihood arising from the observed sequence of exposure events. The third and fourth lines represent the contribution to the likelihood of the sojourn times in class E and I respectively for the exposed individuals.

For mathematical clarity, we have so far discussed a general case where the population density along the circumference $\sigma (l|r,\hat{s})$ is assumed to be continuous. In practice, however, the data of population density over a study area is often provided in a discrete form, mostly on the grid level [26] (see also Fig 1). We describe how this special case may be handled practically in S1 Text and S1 Fig.

### Statistical inference

We conduct Bayesian inference of partially observed epidemics using the process of data augmentation supported by Markov chain Monte Carlo methods [4, 27–29]. Given observed partial data ***y***, including times of symptom onset and death times, the inference involves sampling from the joint posterior distribution *π*(**Θ**, ***z***|***y***) ∝ *L*(**Θ**; ***z***)*π*(**Θ**), where ***z*** represents the complete data and *π*(**Θ**) represents the prior distribution of model quantities, such that the complete *z* is reconstructed, or ‘imputed’. We use weak uniform priors *U*(0, 100). It is noted that, in analyzing the Ebola outbreak data (see *Example: Application to the Ebola Outbreak Data*) where ***z*** = (**E**, **I**, **R**, **G**, ***ψ***), other than the parameters in **Θ** = (*α*, *β*, *a*, *b*, *c*, *η*, *ω*), the exposure times **E** and the sources of infections ***ψ*** (i.e. the transmission tree) are unobserved and are also to be inferred [4, 27].

## Results

### Validation of model inference

**Fig 3:**
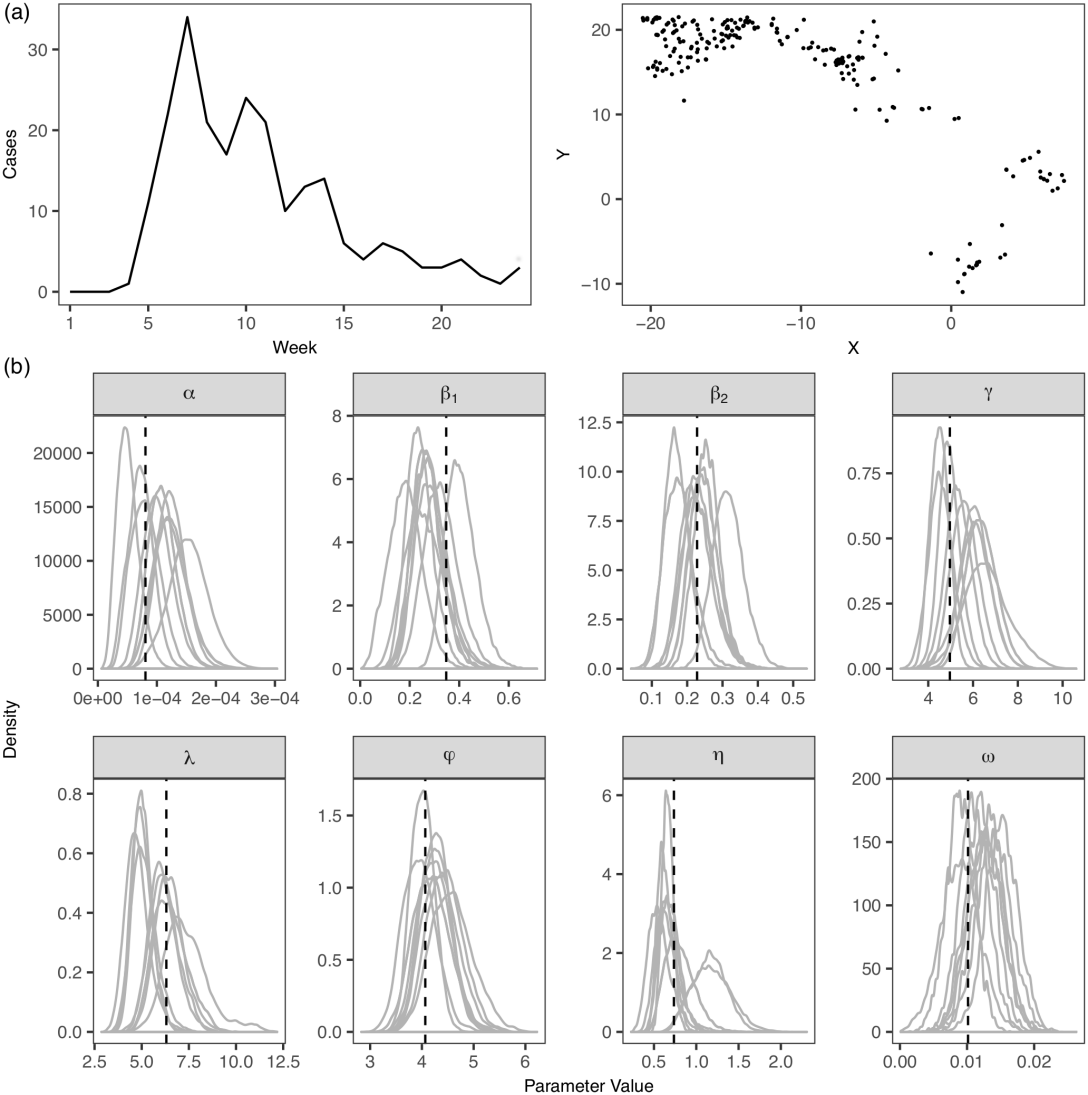
Validation of model inference. (a) Temporal and spatial distributions of the cases in an epidemic simulated from our model; noted that the spatial coordinates are converted to distance (kilometers) relative to the point where Lat = 8.3 Lon = -13.1; (b) Model parameters used for simulating 10 independent epidemics from our model are indicated by the dotted lines; the inferred posterior distributions of the model parameters are also shown.

In this section we test our methodology using simulated datasets. 10 independent epidemics are simulated from the model described in *Model and Methods*, parameterized by a set of model parameter values arising from fitting to an Ebola outbreak data (see *Example: Application to the Ebola Outbreak Data*). The same observation period, geographical area and population density as the Ebola data are considered. Fig 3a shows an exemplar simulated epidemic. Similar to the application to the Ebola outbreak data, we also consider age-specific baseline transmissibility of an infectious individual, i.e. *β*_1_ for age less than 15 and *β*_2_ for age greater than or equal to 15. Subsequently, we fit our model to each of the simulated epidemics and obtain the posterior samples of the model parameters. Fig 3b suggests that the model parameters can be accurately estimated from the corresponding inferred posterior distributions which cluster around the true parameter values. We also test with another set of simulated datasets in which we assume a different distribution of population density, suggesting the similar accuracy in parameter estimations (S2 Fig).

### Comparison with individual-based SEIR model

Conventional SEIR models, which require an *explicit* account of the contact network among *all subjects*, have proven to be useful in studying patch-level level disease transmission (Fig 2a), e.g. among farms, towns and cities [7, 27]. While these models are not theoretically restricted to the patch-level, they are often computationally challenging for individual-level data arising from moderate- to large-size populations. Although these models are not preferable in the scenario considered in the paper, they may be utilized to generate ‘reference’ epidemics that can be subsequently used for further assessing our framework.

**Fig 4:**
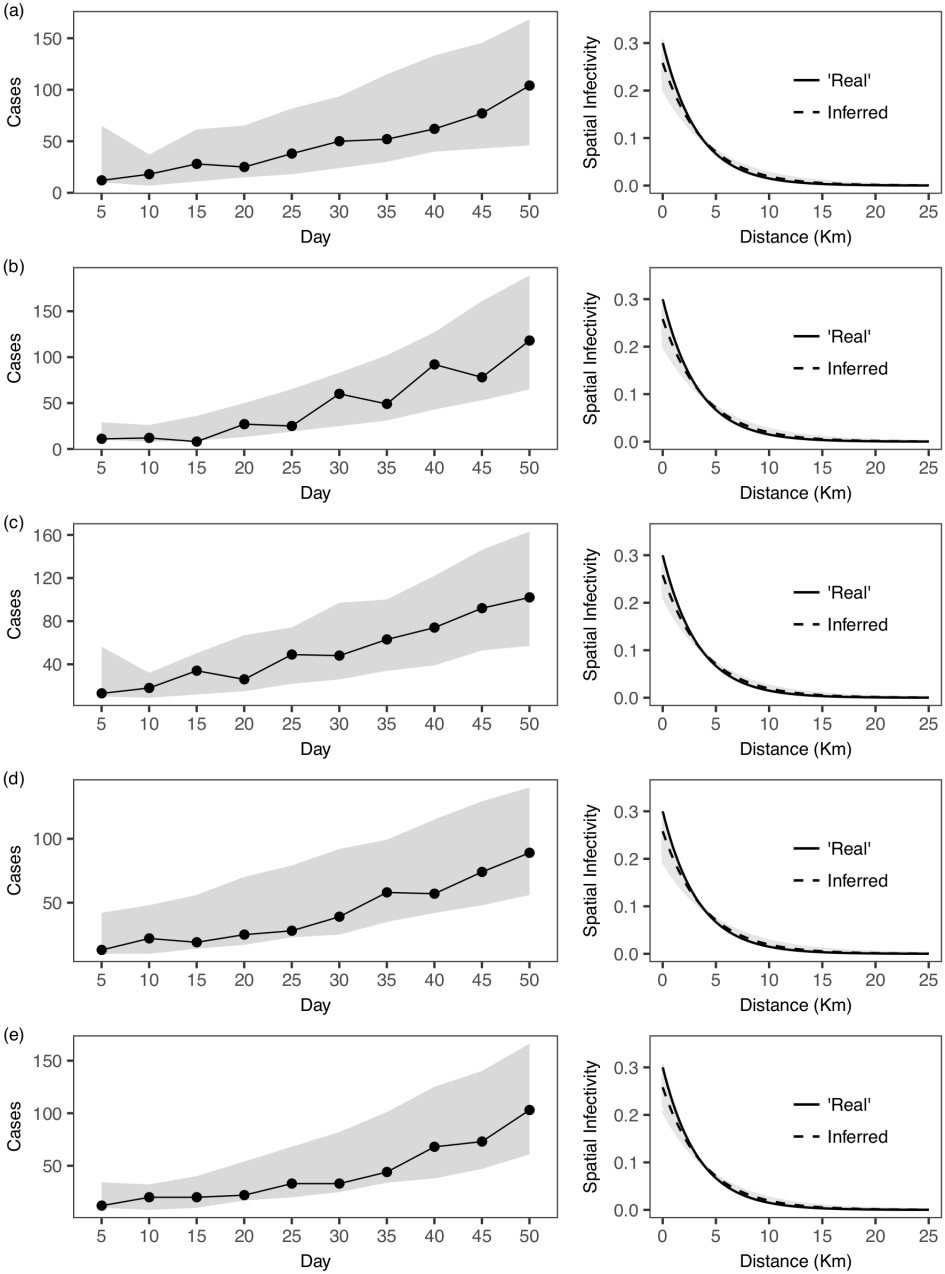
Comparison with the individual-based SEIR model. ‘Real’ epidemics (black dots and line) are first simulated from an individual-based SEIR model (see also S1 Text). Subsequently, our proposed framework is fitted to the simulated epidemics. The fitted model is then used in predictive mode to simulate epidemics (95% C.I. in grey). We first compare the incidence with 5-day intervals between the ‘real’ epidemics (from SEIR) and the forward-simulated epidemics (from our fitted model). We also compare the (normalized) ‘real’ distance-dependent spatial infectivity (solid line), with that inferred from our framework (dotted line) using the posterior parameter means. 500 random set of parameter values from the posterior distribution are drawn and their corresponding inferred spatial infectivity (grey lines) are also shown. Results from 5 independent simulations are shown (a)–(e).

In this section we perform simulation studies to understand how our framework may capture the temporal and spatial dynamics of the epidemics generated from the SEIR model. We focus on simulations from an individual-based and susceptible-explicit SEIR model, in a heterogeneous landscape, that give rise to epidemics in which around 5% of a study population becomes infected (within 50 days of the initial infection). We note that the prevalence we consider is significantly higher than that found in the recent Ebola outbreak and matches more closely other, more transmissible viruses such as influenza [30]. We consider simpler scenarios with no control measures and known latent period distribution. Details of the SEIR model are given in S1 Text. Fig 4 suggests that our framework can capture key temporal and spatial dynamics of the epidemic simulated from the individual-based SEIR model. Similar results are observed in testing with another set of simulated epidemics (S3 Fig), in which we consider a scenario with a different population density distribution and a fatter tail in the spatial transmission distance.

We also perform a comparison between the run-time of our model inference and that of performing full individual-based SEIR model inference, which suggests that ours can be about 780 times faster (see also S1 Text).

### Example: Application to the Ebola outbreak data

#### Ebola outbreak data

We also deploy our methodology to a dataset describing Ebola transmission in the community, collected from the Safe and Dignified Burials (SDB) programme conducted by the International Federation of Red Cross (IFRC), between Oct 20, 2014 and March 30, 2015 in Western Area (which comprises the capital Freetown and its surrounding area) in Sierra Leone. The dataset contains mobile-phone-reported GPS locations of where the bodies of 200 fatalities tested positive for Ebola (Fig 1). Age, sex, time of burial (which was usually performed within 24h of death) and symptom-onset time were also recorded. Population density data were obtained from [26].

The same dataset was previously analyzed in [4], using a transmission-network-based (partial-likelihood) approach (Fig 2b). Although it was shown that such an approach is useful for inferring key epidemiological quantities (e.g. basic reproductive number *R*_0_) and sampling the summary topology of the transmission tree among the observed cases, it does not consider the general (susceptible) population—as a result it cannot be used to establish a relation between infections and the general population, something that is necessary if more general model-based forward predictions are to be made. In this section we compare our results with the findings of the previous analysis. In particular, we show how a model-based, forward prediction may be made using our methodology. In this section we consider age-specific baseline transmissibility, i.e. *β*_1_ for age less than 15 and *β*_2_ for age greater or equal to 15. In the forward simulation, the distribution of age (group) for a new infection is assumed to be the empirical distribution of the age groups of the observed data (which may also be estimated from more general demographic data).

### Model estimates

#### Reproductive number

**Fig 5:**
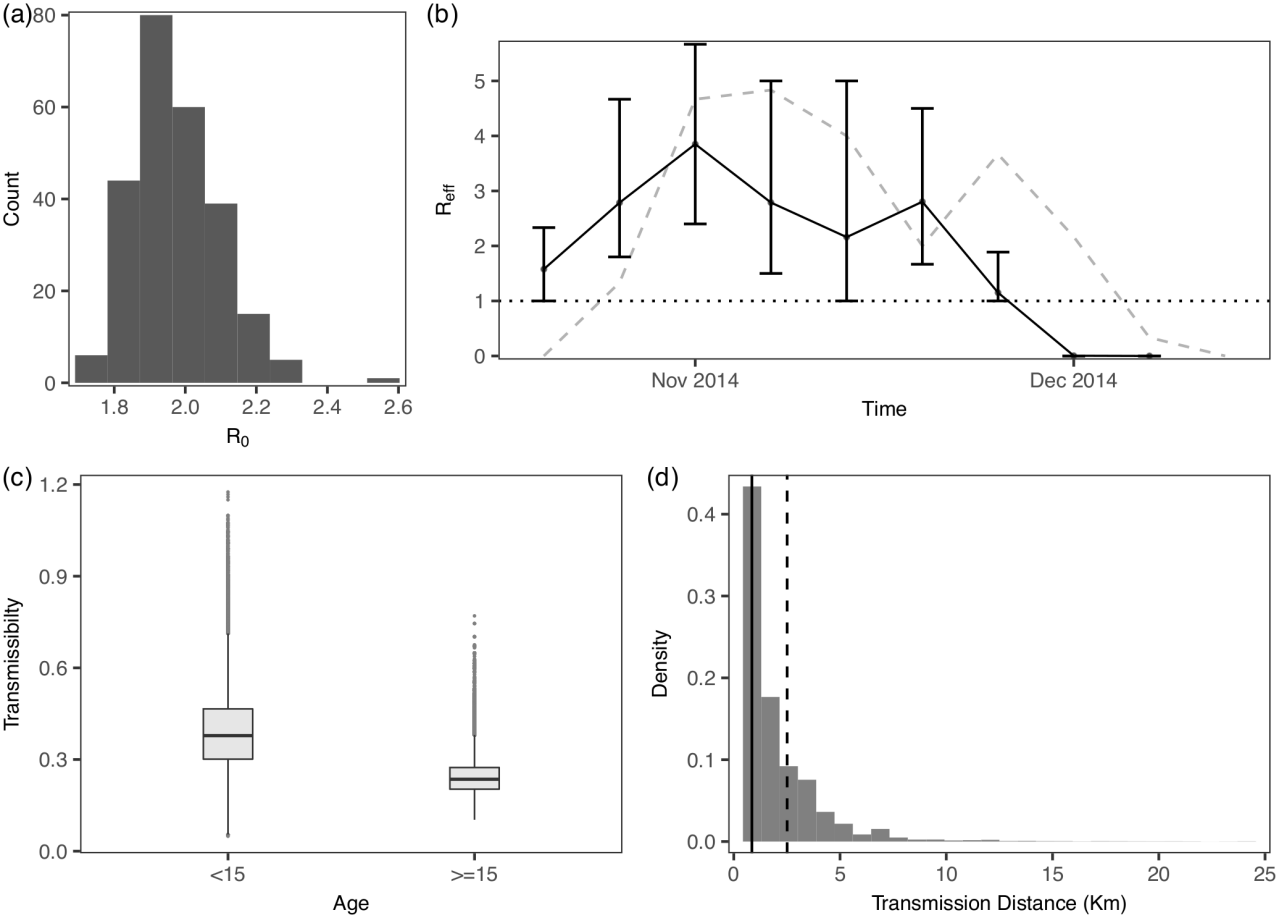
Model estimates for the Ebola dataset. (a) The posterior distribution of *R*_0_. (b) The posterior distribution of *R*_*eff*_; median values are connected by the dots and lines, and the 95% C.I. are indicated by the bars. The observed epidemic curve, scaled by dividing by 6, is superimposed (dotted grey line). (c) The posterior distributions of age-specified *β*. (d) The posterior distribution of distance of transmission with median value indicated by the solid black line; the dotted line represents the median value estimated in [4].

A key epidemiological parameter is the so-called basic reproductive number *R*_0_, or the time-dependent variant effective reproductive number *R*_*eff*_, which quantifies the average number of secondary cases generated by a given infection [31–33]. In our framework the transmission tree is imputed, from which we can compute *R*_0_ and *R*_*eff*_ as summary statistics. We estimate *R*_0_ to be 2.0 with 95% C.I. [1.8, 2.2] (Fig 5a), which is slightly lower than the estimate 2.39 in [4]. The estimate of *R*_*eff*_ (Fig 5b) is also broadly consistent with that found in [4] and in the literature (e.g., [31]). It is also noted that degree of super-spreading was commonly characterized using a dispersion parameter *k* summarized from the transmission tree [4, 34, 35]. Estimated values for *k* are 0.47 and 0.37, using our methodology and that used in [4] respectively, both indicating significant super-spreading (*k* < 1), albeit to a lesser extent (i.e. higher *k*) here.

#### Age-specified transmissibility and distance of transmission

In [4], it was found that certain age groups tend to be more transmissible—in particular, infected individuals younger than 15 or older than 45 years. Using our methodology, although we find no significant difference among subgroups of those older than 15, there is still clear evidence that cases less than 15 tend to be most transmissible (Fig 5c). In fact, this age group was found to be the most transmissible in [4]. The median distance of transmission is estimated to be 0.85km [0.01, 6.15], which is about one third of the estimate 2.51km found in [4]. Such a discrepancy may reflect the fact that the heterogeneous (susceptible) population is now taken into account, with the presence of many disease-free areas reducing the likelihood of long-range transmission. A shorter distance of transmission may also be potentially more accurate, considering that the pathogen may have spread predominantly by caring within the community, e.g., through family contacts [36]. Estimates of other model parameters are given in S1 Table, showing broad consistency with the literature [4, 37, 38]. However, it is noted that our estimate of mean infectious period is lower than from cases detected within the clinical care system (e.g. mean infectious period 8d estimated for patients who received clinical care [39]). As discussed in [4], this discrepancy potentially highlights systematic differences between community-based cases and cases notified in clinical care systems, where community-based cases may have progressed more rapidly.

### A more general model prediction

**Fig 6:**
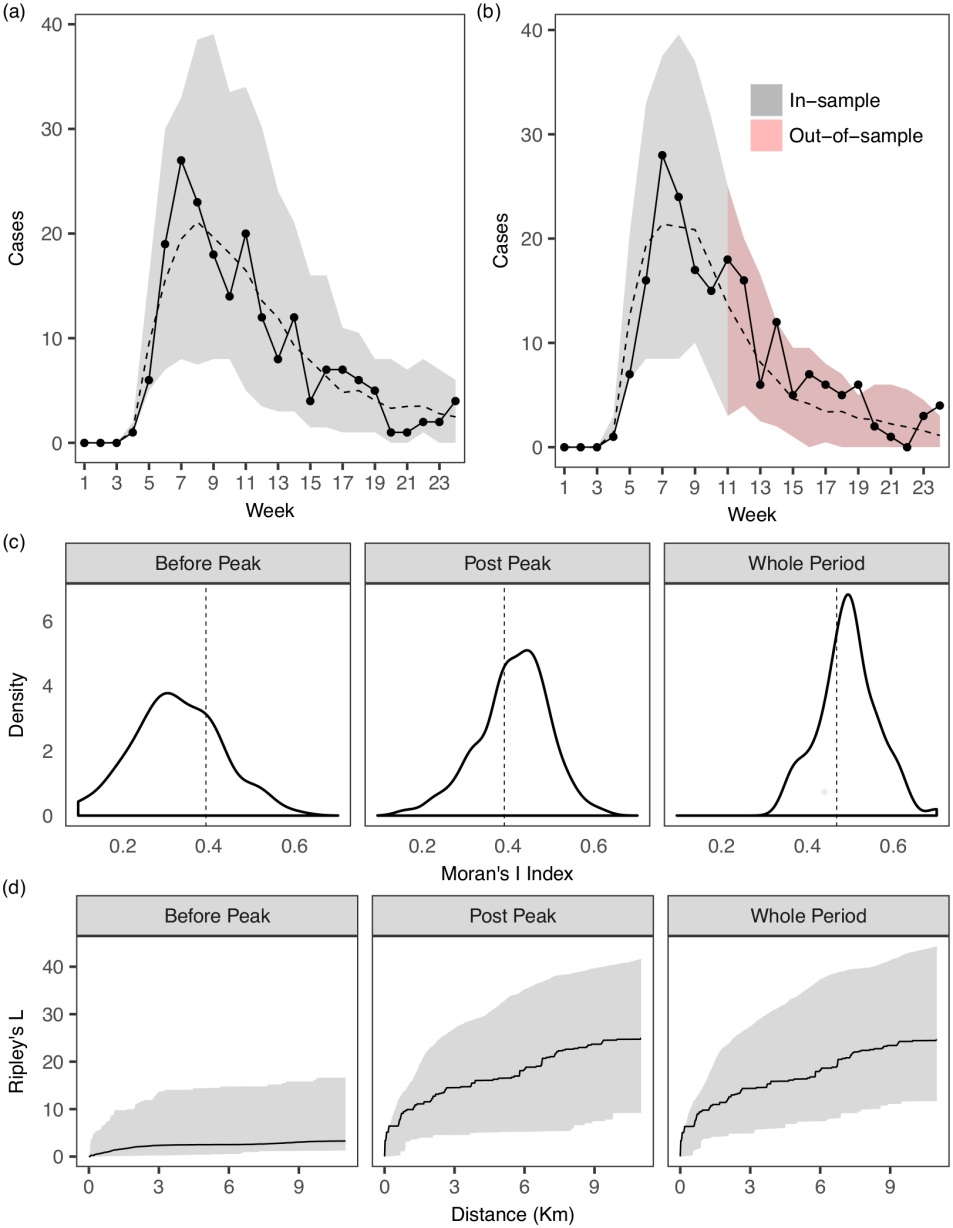
Posterior predictive distributions of temporal and spatial summary statistics of epidemics forward simulated from the estimated model. (a) One-epidemic ahead in-sample model prediction. The observed epidemic is indicated by the dots and the line. 95% C.I. of the simulated epidemics at each week are indicated by the grey bands. Dashed line represents the median values of the simulated epidemics. (b) One-epidemic ahead in-sample and out-of-sample model prediction. We first estimate the model parameters using data from the first half of the epidemic duration (week 1 to week 11) and re-simulate from the estimated model from the beginning to the end of the epidemic duration (week 24). (c) Measure of global spatial autocorrelation using Moran’s I index (Refs. [7]) which ranges from -1 to 1 (a value close to 1 indicating strong clustering and close to -1 indicating strong dispersion), applied to epidemics before and after peak. The index corresponding to the observed epidemic is indicated by the dotted line. (d) Measure of clustering/dispersion using Ripley’s K, or its transformation Ripley’s L [40, 41]. Compared to global measures such as the Moran’s I, this function determines clustering/dispersion of point data over *a range* of distances (see S1 Text for more details), *without* requiring certain aggregation of the points, hence representing a more powerful and informative measure for our context. We consider and compute the L function using the the R package *spatstat* [42]. The measure corresponding to the observed epidemic is indicated by the solid line, along with the 95% C.I. of the simulated epidemics enclosed in the grey band. They indicate that the spatial clustering/dispersion of observed (point) data are captured reasonably well.

In contrast to a transmission-network based approach [4], our framework establishes a relation between infections and the general (susceptible) population. Specifically, it proposes a mechanism for how a new infection, beyond the set of observed infected individuals, can arise among the general (susceptible) population. This in turn allows us to perform a more general forward simulation without conditioning on the set of observed cases. Fig 6 shows the (posterior predictive) distributions of some temporal and spatial summary statistics of the epidemics simulated from the estimated model, from which it can be discerned that the model can generate epidemics that are consistent with the observed one. We also show out-of-sample predictivity for the epidemic curve for the second-half of the epidemic duration (Fig 6b). It is noted that in assessing the spatial fit, beside using a relatively crude global measure (i.e. Moran’s I index (Refs. [7])), we also consider Ripley’s L function [40, 41] which is much more informative for characterizing clustering/dispersion of *point* data.

## Discussion

More precise individual-level spatio-temporal data have become increasingly available in recent years due to the advent of ‘digital epidemiology’ [1]. One key challenge is how we may extract maximal information from such data, especially through concurrent development of new statistical methods, as existing approaches suffer from certain limitations (see Introduction). In particular, as SEIR-type models can be computationally challenging for individual-level spatio-temporal data, new frameworks are needed to accommodate such data in a mechanistic manner. The recent Ebola outbreak in West Africa (2014-2016) highlights the need, in particular, for a statistically sound and computationally efficient framework that is both able to integrate individual temporal and spatial information and, more importantly, perform a more general forward prediction which needs to take into account the general susceptible population [4].

In this paper, we have proposed a novel mechanistic framework to address the research gap. Application to the Ebola outbreak data shows broad consistency of key epidemiological quantities with a previous analysis using a transmission-network-based partial-likelihood approach [4], despite a significantly lower, and potentially more accurate [36], median value of estimated distance of transmission (0.85km vs 2.51km). We have shown that our methods can be used in predictive mode to simulate epidemics (among the general population) that are consistent with the observed temporal and spatial patterns of the real outbreak, enabling a more general epidemic predictive framework. We also tested our model inference using simulated examples. Our model was also compared to the more explicit (but computationally challenging) individual-based SEIR model, showing that our model can be a reasonable and computationally-efficient surrogate.

There are a few simplifying assumptions made in our paper. For example, we have focused on epidemic outbreaks that are mainly attenuated by a time-varying transmissibility e.g. due to controls or seasonal changes of transmissibility. Should susceptible depletion play a key role in attenuating the epidemics, our framework may be modified accordingly—e.g., for a given region, adding a component that specifies the decreased likelihood of occurrence of new infections with increased density of existing infections, to mimic the effect of susceptible depletion. Nevertheless, the effect of susceptible depletion may only be significant on a very local scale such as that of the individual household. Moreover, it does not appear to be a determining factor in controlling the recent Ebola outbreak, at least on the ‘global’ scale [17] (Fig 6). We have considered random movement patterns of infectious individuals that may be reasonably abstracted by a monotonically decreasing density function [23, 24]. For future work, this assumption may be relaxed to model more complicated scenarios, such as spread of splash-dispersed fungal pathogens [43] in which the spreading distance may also depend on the susceptible population. In this case, one may modify the density for the distance by also taking into account the distribution of susceptible population in the annuli along the radius of the circle centered at a particular source of infection.

The transmission rate of an infectious case in our model is independent of the (local) susceptible population density. This assumption may be relaxed to allow for more “localized” transmission rates. For example, a model taking into account the heterogeneity of the susceptible population more explicitly may be obtained by allowing the infection rate for each case to be dependent on the local density of susceptibles by taking an appropriate weighted average of the latter with respect to the kernel function, at the expense of increased computational complexity. When spatial heterogeneity is present at a scale that is fine with respect to the range of transmission, then such an average may exhibit little variability over cases. Nevertheless, we note the ability of our approach to identify a kernel that matches that identified when the full SEIR model is fitted. Moreover, our model appears to be reasonable for the case of the Ebola outbreak (Fig 6).

We have considered scenarios that the entire population is susceptible, an assumption which generally holds for newly emerging infections. Vaccination, for instance, decreases the proportion of susceptibles among the general population, and has an important impact on the geographical spread of viruses (e.g. [44]). The effect of vaccination can be readily incorporated by our framework, for example, by reducing the (effective) susceptible population proportional to the vaccination rate in a particular region. The Ebola dataset we analyzed is likely to be subject to underreporting, which may have resulted in, for example, a biased (lower) estimate of the degree of superspreading [4]. Future work which takes into account the underreporting explicitly may be considered. We hope that our proposed framework can provide an essential step for the systematic modelling of the increasingly available individual-level disease data.

## Supporting information

S1 Text Supplementary information. (a) Simulation algorithm for our proposed individual model. (b) Practical implementations in dealing with the grid-nature of population density data. (c) Detailed procedures for simulating epidemics from the individually-based SEIR model for comparing it with our proposed framework (see also section *Comparison with Individual-based SEIR Model*). (d) Speed gain in our model inference by comparing to individual-SEIR model inference. (e) Supplementary information for Ripley’s L function which was used to summarize the spatial clustering of the observed and model-simulated Ebola epidemics.
(PDF)

S1 Fig Dealing with the grid nature of population density data. Intersecting with the (dotted) grid lines, the circumference of the circle with radius *r* centered at a source of infection is divided into many arcs. Each arc and the grid it belongs to has a homogeneous population density. One arc segment (in grey), for example, has arc length Δ*l* and arc segment angle Δ*θ*.
(PDF)

S2 Fig Validation of model inference. Here we consider a different distribution of population density compared to the one used for the Ebola dataset in the main text. In particular, we consider a random shuffling of the original grids of population density. (a) Temporal and spatial distributions of the cases in an epidemic simulated from our model; noted that the spatial coordinates are converted to distance (kilometers) relative to the point where Lat = 8.3 Lon = -13.1; (b) Model parameters used for simulating 10 independent epidemics from our model are indicated by the dotted lines; the inferred posterior distributions of the model parameters are also shown.
(PDF)

S3 Fig Comparison with the individual-based SEIR model. Here we consider a different distribution of population density compared to the one used for the Ebola dataset in the main text. In particular, we consider a random shuffling of the original grids of population density. We also allow for a fatter tail of spatial transmission distance. ‘Real’ epidemics (black dots and line) are first simulated from an individual-based SEIR model (see also S1 Text). Subsequently, our proposed framework is fitted to the simulated epidemics. The fitted model is then used in predictive mode to simulate epidemics (95% C.I. in grey). We first compare the incidence with 5-day intervals between the ‘real’ epidemics (from SEIR) and the forward-simulated epidemics (from our fitted model). We also compare the (normalized) ‘real’ distance-dependent spatial infectivity (solid line), with that inferred from our framework (dotted line) using the posterior parameter means. 500 random set of parameter values from the posterior distribution are drawn and their corresponding inferred spatial infectivity (grey lines) are also shown. Results from 5 independent simulations are shown (a)–(e).
(PDF)

S1 Table Supplementary table. Estimates of model parameters in fitting our framework to the Ebola dataset.
(PDF)
