# Supplementary material for: A mechanistic spatio-temporal framework for modelling individual-to-individual transmission—With an application to the 2014-2015 West Africa Ebola outbreak
Source: PLoS Comput Biol. 2017 Oct 30;13(10):e1005798. doi: 10.1371/journal.pcbi.1005798 (PMC5679647; doi:10.1371/journal.pcbi.1005798)
Supplement: S1 Text — (a) Simulation algorithm for our proposed individual model. (b) Practical implementations in dealing with the grid-nature of population density data. (c) Detailed procedures for simulating epidemics from the individually-based SEIR model for comparing it with our proposed framework (see also section Comparison with Individual-based SEIR Model). (d) Speed gain in our model inference by comparing to individual-SEIR model inference. (e) Supplementary information for Ripley’s L function which was used to summarize the spatial clustering of the observed and model-simulated Ebola epidemics. (PDF) [file pcbi.1005798.s001.pdf]

S1 Text

Simulation Algorithm

Here we describe an algorithm for simulating epidemics from our proposed individual model. Consider the situation where there is one infectious individual at current time t (i.e. the time of this individual transits from class E to class I) among an entirely susceptible population. The simulation proceeds as follows:

1. Draw a waiting time $\triangle t_I$ in class I from a specified infectious period distribution, and set the time of recovery $t_R = t + \triangle t_I$.
2. Let t_{next} be the earliest time in the union of t_I and t_R (those greater than t) of all currently infectious individuals.
3. The time of next infection t_E is drawn as the first event in a non-homogeneous Poisson process with a time-varying rate $\beta(t)$ (defined in the main text). There are a number of ways of simulating from a non-homogeneous Poisson process − for example, the thinning algorithm [1] which essentially samples the next event time t_E from a homogeneous Poisson process with the baseline rate β and accepts that with probability $\beta(t_E)/\beta$. Note that when there are $n > 1$ currently infectious individuals, the acceptance probability would become $\frac{\sum_j^n \beta_j(t_E)}{n\beta}$.
4. If $t_E < t_{next}$, set $t = t_E$.
5. If $t_E \geq t_{next}$, set $t = t_{next}$, and resimulate t_E as described in Step 3.
6. After t_E is sampled, select the source of this infection uniformly among currently infectious individuals. This new infection is then placed at distance r and angle $\Delta\theta$ from the selected source according to the density $g(G = (r, \Delta\theta); \eta, \hat{\boldsymbol{s}})$ specified in details in the main text.
7. Draw t_I the time of becoming infectious and t_R the recovery timefor this new infection using specified waiting time distributions.
8. Repeat the above steps until a stopping criterion (e.g. $t > t_{max}$) is reached.

On the Grid-nature of Population Density Data

In the main text, for mathematical clarity, we have discussed a general case where the population density along the circumference $\sigma(l|r, \hat{\boldsymbol{s}})$ is assumed to be continuous. In practice, however, the data of population density over a study area is often provided in a discrete form, mostly on the grid level. This results in a piecewise constant density of the angle θ, with the precise location of change-points in this density depending on how the 'grids' and the 'circle' intersect (see Fig. **??**). To derive this density, it is necessary to compute the probability masses of the arcs (which is proportional to the density of the corresponding angles) that comprise the whole circumference, where population density is homogeneous/constant on each arc (and in the grid it belongs). Here we describe an algorithm for computing $\sigma(l|r, \hat{\boldsymbol{s}})$ given the grid-level population density. First of all, using basic plane and line geometry, we solve for the coordinates of the intersection points between the grid lines and the circle (Fig. **??**). Then, for each intersection point (x_i, y_i) we solve for the angle θ_i measured from the first quadrant by

$$\theta_i = \arctan(\frac{y_i - y_s}{x_i - x_s}), \tag{1}$$

where (x_s, y_s) is the coordinate of the source of infection (the center of the circle). Let $(\theta_{(1)}, \theta_{(2)} ....)$ be the vector of ordered angles, and let $\Delta\theta_i = \theta_{(i+1)} - \theta_{(i)}$, for $i = 1, 2, ...$ The probability mass of an arc segment with $\Delta\theta_i$ is then proportional to

$$\sigma_{\Delta l_i} \times \Delta l_i, \tag{2}$$

where Δl_i is the arc length corresponding to $\Delta\theta_i$, and $\sigma_{\Delta l_i}$ is the constant population density of the grid-cell in which the arc lies.

It is worth noting that infected individuals emerge in continuous space regardless the form of the population density data.

Simulating from Individual SEIR Model for Comparison

We describe how we simulate epidemics from the individually-based SEIR model described in the main text section *Comparison with Individual-based SEIR Model.* For the sake of computational efficiency, we consider an initially susceptible population with population size $N = 10000$. The geographical locations of these N individuals were randomly assigned accordingly to a normalized population density across the study area similar to the Ebola dataset. An epidemic was assumed to be initiated by an index case and progressed according to the conventional spatial-temporal SEIR epidemic model [2]. In contrast to our proposed framework, the conventional SEIR model requires an *explicit* specification of the infectious challenge presented to each susceptible individual. Specifically, a susceptible individual j becomes exposed during $[t, t+dt)$ with probability

$$p(j,t) = \{\alpha' + \beta' \sum_{i\in\xi_I(t)} K(d_{ij},\kappa')\}dt + o(dt), \tag{3}$$

where α' represents a primary infection rate and β' is the contact parameter. The term $K(d_{ij},\kappa')$ characterises the dependence of the infectious challenge from infective i to j as a function of distance d_{ij} and is known as the spatial kernel function. We assume that $K(d_{ij},\kappa') = exp(-\kappa' d_{ij})$. It is noted that $K(d_{ij},\kappa')$ does not have to be a proper density function. Similar to our proposed model, an infected individual is assumed to spend random times in class E and I, modelled by $Gamma(\gamma,\lambda)$ and $Exponential(1/\varphi)$ distributions respectively. We focus on simulations that give rise to epidemics in which around 5% of a study population becomes infected (in 50 days). Specifically, we set $\alpha' = 2.5\times10^{-5}$, $\beta' = 1.8\times10^{-4}$, $\kappa' = 0.3$, $\gamma = 3$, $\lambda = 1$ and $\varphi = 7$. It is noted that, as the conventional SEIR model and our model have very distinct structures (and hence in general imply differing interpretations of their model parameters), they are therefore compared based on commonly agreed key 'model outcomes' (see main text).

Speed Gain in Comparing to Individual-SEIR Model Inference

We also fit the full individual-based SEIR model to one of the simulated datasets described in *Simulating from Individual SEIR Model.* It is noted that this will require an explicit consideration of each individual (among the population with $N = 10000$) in the model fitting and often require a large amount of recalculations of likelihood during the MCMC sampling − for example, at proposing a new value of κ', the spatial infectivity between all infectious-susceptible pairs must be recalculated. As the purpose here is to compare the computational efficiency, we only record and compare the run-time for 1000 MCMC iterations both in performing the full individual-based SEIR model inference and performing inference of our model. A comparison, in which both model fittings were conducted using similar C++ codes and data structures, shows a significant speed gain in fitting our model (3 vs 2340 seconds).

Ripley's K and L Function

Ripley's K function [3, 4] is similar to Moran's I function but is able to characterize clustering/dispersion of point patterns at *multiple* distances. Theoretically it is defined as

$$K(r) = \lambda^{-1}\mathbf{E}[\text{number of points within distance r of a randomly chosen point}], \tag{4}$$

where λ is the density of points per unit area. We consider a common transformation of Ripley's K, Ripley's L function $L(r) = \sqrt{K(r)/\pi}$. We compute the estimated $L(r)$ using the function *Linhom*$(\cdot)$ provided in the R package *spatstat*[5], on a range of distance $[0, r_{max}]$. This function also determines

a sensible default of the maximum distance r_{max}, by taking into account the increasing estimation uncertainty at larger distances. It is noted that the estimated $L(r)$ are often compared to those computed from data generated from a null model (i.e. a Poisson point process), in which clustering/dispersion are characterized relative to the null model. In this paper, however, it is the comparison of values of $L(r)$ computed from the model simulated data and the observed data is of interest.

References

[1] Lewis PA, Shedler GS. Simulation of nonhomogeneous Poisson processes by thinning. Naval Research Logistics (NRL). 1979;26(3):403–413.

[2] Lau MS, Marion G, Streftaris G, Gibson G. A systematic Bayesian integration of epidemiological and genetic data. PLoS Comput Biol. 2015;11(11):e1004633.

[3] Ripley BD. The second-order analysis of stationary point processes. Journal of applied probability. 1976;13(02):255–266.

[4] Ripley BD. Statistical inference for spatial processes. Cambridge university press; 1991.

[5] Baddeley A, Turner R, et al. Spatstat: an R package for analyzing spatial point patterns. Journal of statistical software. 2005;12(6):1–42.
