# Supplementary figures and images for: A mechanistic spatio-temporal framework for modelling individual-to-individual transmission—With an application to the 2014-2015 West Africa Ebola outbreak
Source: PLoS Comput Biol. 2017 Oct 30;13(10):e1005798. doi: 10.1371/journal.pcbi.1005798 (PMC5679647; doi:10.1371/journal.pcbi.1005798)

S1 Figure

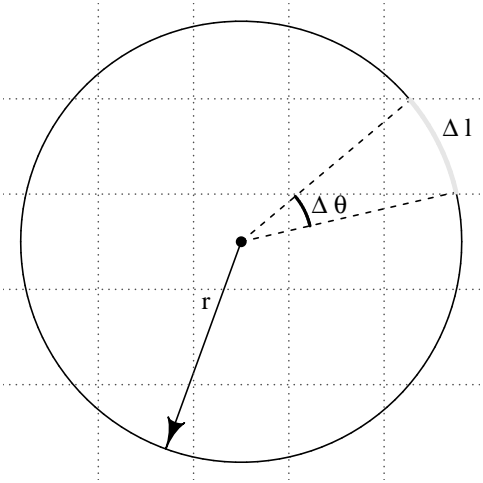

Supplement: S1 Fig — Intersecting with the (dotted) grid lines, the circumference of the circle with radius r centered at a source of infection is divided into many arcs. Each arc and the grid it belongs to has a homogeneous population density. One arc segment (in grey), for example, has arc length Δl and arc segment angle Δθ. (PDF) [file pcbi.1005798.s002.pdf]

# S2 Figure

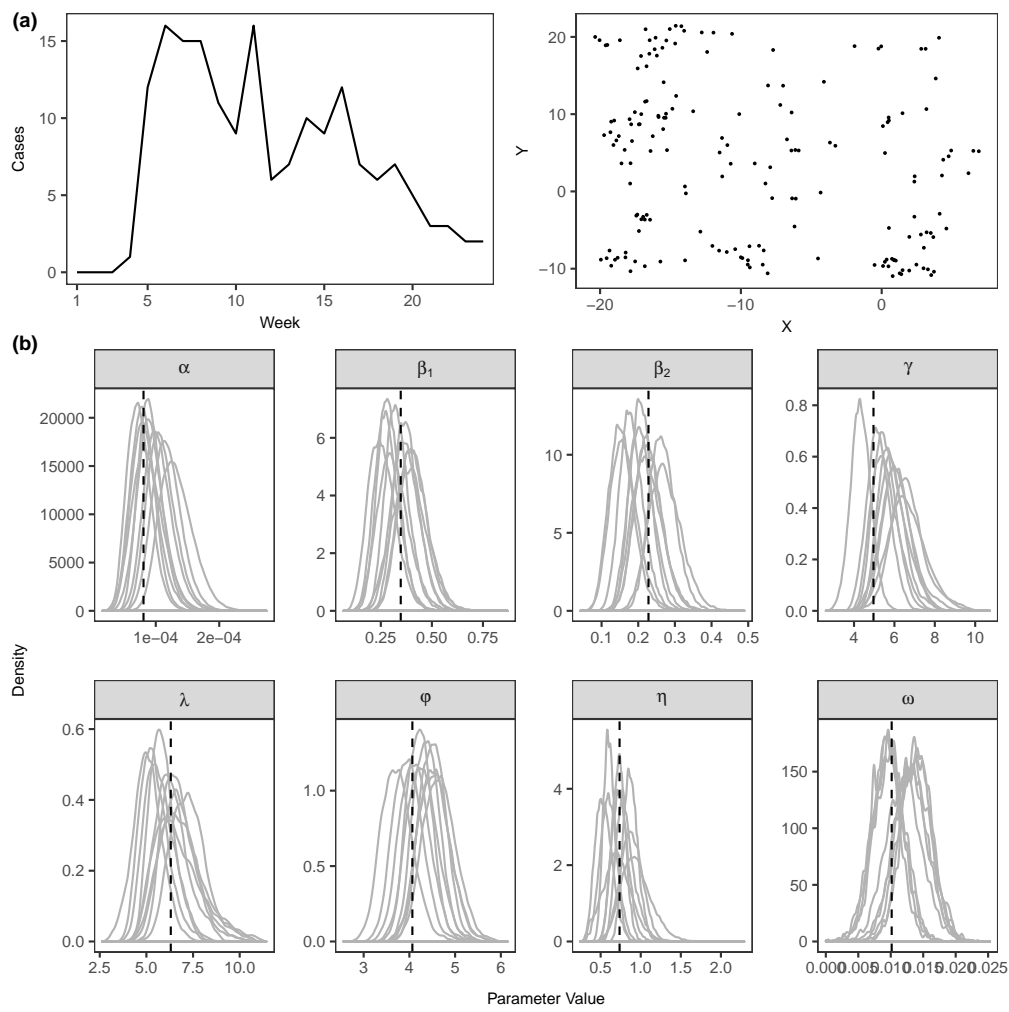

Supplement: S2 Fig — Here we consider a different distribution of population density compared to the one used for the Ebola dataset in the main text. In particular, we consider a random shuffling of the original grids of population density. (a) Temporal and spatial distributions of the cases in an epidemic simulated from our model; noted that the spatial coordinates are converted to distance (kilometers) relative to the point where Lat = 8.3 Lon = -13.1; (b) Model parameters used for simulating 10 independent epidemics from our model are indicated by the dotted lines; the inferred posterior distributions of the model parameters are also shown. (PDF) [file pcbi.1005798.s003.pdf]

# S3 Figure

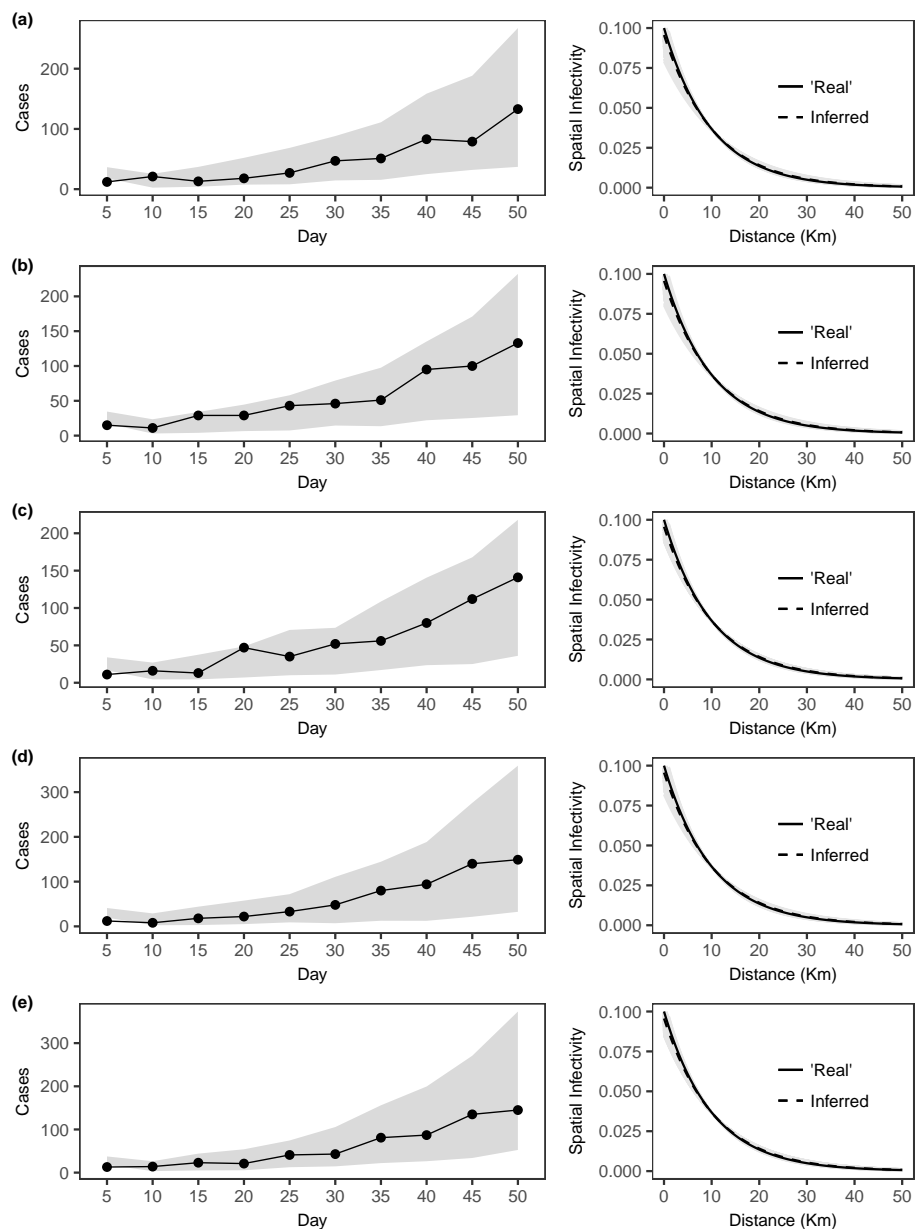

Supplement: S3 Fig — Here we consider a different distribution of population density compared to the one used for the Ebola dataset in the main text. In particular, we consider a random shuffling of the original grids of population density. We also allow for a fatter tail of spatial transmission distance. ‘Real’ epidemics (black dots and line) are first simulated from an individual-based SEIR model (see also S1 Text). Subsequently, our proposed framework is fitted to the simulated epidemics. The fitted model is then used in predictive mode to simulate epidemics (95% C.I. in grey). We first compare the incidence with 5-day intervals between the ‘real’ epidemics (from SEIR) and the forward-simulated epidemics (from our fitted model). We also compare the (normalized) ‘real’ distance-dependent spatial infectivity (solid line), with that inferred from our framework (dotted line) using the posterior parameter means. 500 random set of parameter values from the posterior distribution are drawn and their corresponding inferred spatial infectivity (grey lines) are also shown. Results from 5 independent simulations are shown (a)–(e). (PDF) [file pcbi.1005798.s004.pdf]
