# Supplementary material for: A mechanistic spatio-temporal framework for modelling individual-to-individual transmission—With an application to the 2014-2015 West Africa Ebola outbreak
Source: PLoS Comput Biol. 2017 Oct 30;13(10):e1005798. doi: 10.1371/journal.pcbi.1005798 (PMC5679647; doi:10.1371/journal.pcbi.1005798)
Supplement: S1 Table — Estimates of model parameters in fitting our framework to the Ebola dataset. (PDF) [file pcbi.1005798.s005.pdf]

S1 Table

Table S1

Parameter	Median [95% C.I.]
α ($\times 10^{-5}$), the baseline background rate	7.9 [4.73, 12.5]
ω, intervention efficacy parameter	0.01 [0.005, 0.015]
γ, mean of the latent period	4.9 [3.82, 6.25]
λ, s.d. of the latent period	6.12 [4.79, 8.12]
φ, mean of the infectious period	4.05 [3.53, 4.67]
η, parameter of the spatial dispersal density function	0.73 [0.56, 0.95]
